# Reporting of Fairness Metrics in Clinical Risk Prediction Models Used for Precision Health: Scoping Review

**DOI:** 10.2196/66598

**Published:** 2025-03-19

**Authors:** Lillian Rountree, Yi-Ting Lin, Chuyu Liu, Maxwell Salvatore, Andrew Admon, Brahmajee Nallamothu, Karandeep Singh, Anirban Basu, Fan Bu, Bhramar Mukherjee

**Affiliations:** 1 Department of Biostatistics School of Public Health University of Michigan Ann Arbor, MI United States; 2 Department of Epidemiology and Biostatistics College of Human Medicine Michigan State University East Lansing, MI United States; 3 Departments of Biostatistics, Epidemiology, and Informatics and Genetics University of Pennsylvania Philadelphia, PA United States; 4 Division of Pulmonary and Critical Care Medicine Department of Internal Medicine University of Michigan Ann Arbor, MI United States; 5 Institute for Healthcare Policy & Innovation University of Michigan Ann Arbor, MI United States; 6 Department of Medicine School of Medicine University of California San Diego San Diego, CA United States; 7 The Comparative Health Outcomes, Policy, and Economics (CHOICE) Institute School of Pharmacy University of Washington Seattle, WA United States; 8 Department of Biostatistics Yale School of Public Health Yale University New Haven, CT United States

**Keywords:** bias, cardiovascular disease, COVID-19, risk stratification, sensitive features, clinical risk prediction, equity

## Abstract

**Background:**

Clinical risk prediction models integrated into digitized health care informatics systems hold promise for personalized primary prevention and care, a core goal of precision health. Fairness metrics are important tools for evaluating potential disparities across sensitive features, such as sex and race or ethnicity, in the field of prediction modeling. However, fairness metric usage in clinical risk prediction models remains infrequent, sporadic, and rarely empirically evaluated.

**Objective:**

We seek to assess the uptake of fairness metrics in clinical risk prediction modeling through an empirical evaluation of popular prediction models for 2 diseases, 1 chronic and 1 infectious disease.

**Methods:**

We conducted a scoping literature review in November 2023 of recent high-impact publications on clinical risk prediction models for cardiovascular disease (CVD) and COVID-19 using Google Scholar.

**Results:**

Our review resulted in a shortlist of 23 CVD-focused articles and 22 COVID-19 pandemic–focused articles. No articles evaluated fairness metrics. Of the CVD-focused articles, 26% used a sex-stratified model, and of those with race or ethnicity data, 92% had study populations that were more than 50% from 1 race or ethnicity. Of the COVID-19 models, 9% used a sex-stratified model, and of those that included race or ethnicity data, 50% had study populations that were more than 50% from 1 race or ethnicity. No articles for either disease stratified their models by race or ethnicity.

**Conclusions:**

Our review shows that the use of fairness metrics for evaluating differences across sensitive features is rare, despite their ability to identify inequality and flag potential gaps in prevention and care. We also find that training data remain largely racially and ethnically homogeneous, demonstrating an urgent need for diversifying study cohorts and data collection. We propose an implementation framework to initiate change, calling for better connections between theory and practice when it comes to the adoption of fairness metrics for clinical risk prediction. We hypothesize that this integration will lead to a more equitable prediction world.

## Introduction

Prediction models are increasingly prevalent in research and decision-making across a wide swath of fields, from finance to criminal justice to health care, and are a key building block in the practice and translation of precision health. Clinical prediction models, including updated versions of classical models like the Framingham Risk Score and the Gail Model [1,2], can leverage granular levels of multimodal data to optimize the implementation of precision health goals and enable the identification of high-risk individuals. However, potential statistical and historical biases ingrained in these models can impact their accuracy and ethical viability, particularly for populations at risk of discrimination due to sensitive features like race, ethnicity, age, or sex (Textbox 1) [3-6]. These risk prediction algorithms, designed to deliver individualized prevention strategies, are rarely evaluated across broad and coarse demographic categories, including the sensitive features mentioned above. Are these seemingly objective prediction models furthering existing inequities in precision health? Particularly as conversational and generative artificial intelligence tools are further integrated into the clinical prediction models that determine who is at high disease risk, who receives an intervention, or who receives prevention resources [6-8], and as the real-time implementation of these models in a hospital setting becomes more common [6], it is critical that these models are fair, providing accurate and nondiscriminatory predictions.

**Definitions of commonly used sensitive variables (also called sensitive features). Note that the definition of these variables is frequently left ambiguous in practice, raising the possibility for harmfully conflating the social and the biological.**

Sensitive variables and their definition

**Social variables**

Variables are based on an individual’s lived experience. Frequently self-reported.

- **Race**
  A social construct based on perceived physical differences that often acts as a proxy for various social and health consequences of racism in modeling [9]. Frequently paired with ethnicity, though they are not strictly equivalent. Includes no biological information.
- **Ethnicity**
  A social construct based on shared culture, language, geography, religion, and history [10]. Frequently paired with race, though they are not strictly equivalent. Includes no biological information.
- **Gender**
  A social construct that refers to an individual’s self-presentation in society, informed by culture, psychology, and society [11]. Though distinct from sex, this variable often indirectly captures sex’s impact.

**Biological variables**

Variables are based on an individual’s biology.

- **Genetic ancestry**
  Genetic similarities between people due to common ancestors [10]. Distinct from race. Strictly biological.
- **Sex**
  The biological factors used to categorize individuals as male, female, or intersex [11]. Since 2016, it has been a required covariate in NIH-funded research. Though distinct from gender, this variable often indirectly captures gender’s impact.

Algorithmic fairness is closely related to but theoretically distinct from algorithmic bias, another important consideration for assessing model performance. For further discussion of the subtle differences between these concepts, we refer the reader to the nuanced comparisons in [12,13]. We focus on algorithmic fairness in the current paper. Algorithmic fairness is concerned with designing algorithms and artificial intelligence models in a way that minimizes or mitigates bias and ensures fair treatment for all individuals or groups affected by the model [12,13]. Achieving algorithmic fairness requires careful consideration of the design, development, and deployment of models, including the selection of appropriate training data and the incorporation of fairness metrics to evaluate model performance on test data.

In recent years, several metrics have been introduced to evaluate the fairness of prediction models [14], alongside various coding toolboxes for their computation and implementation [15,16]. These metrics differ from common prediction metrics measuring discrimination, accuracy, and calibration, which measure overall model performance [12]. These fairness metrics typically aim to assess differences in model predictions (
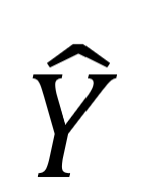
) for a binary decision outcome *Y* (eg, treatment or no treatment) for different values of a sensitive categorical variable *S* (eg, male or female sex)*,* thus giving a numerical sense of how fair or unfair a model may be. The common practice is to provide point estimates of a fairness metric; while fair inferential methods have been proposed [17], uncertainty quantification and interval estimation are not yet widely adopted in fairness research. Some of the most cited and used fairness metrics are listed and summarized in Table 1.

Though quite simple in terms of their definition, such metrics can shed light on otherwise unseen disparities in a model or dataset. For these metrics to produce meaningful results, a clear interpretation of the predictor and sensitive variables used is required in addition to how they affect the outcome of interest *Y*. For the sensitive features that fairness metrics usually seek to assess, it is not always simple to explain what they represent. Sensitive features often have multiple definitions and interpretations, particularly when it comes to what is biological versus what is socially constructed. This ambiguity is especially important for sex and race or ethnicity, 2 of the most used variables in learning algorithms. Sex is a biological variable, but it will likely capture effects from an individual’s gender, information that might be the actual cause behind the sex variable’s recorded influence on an outcome of interest. In the case of race and ethnicity, these variables are often self-reported, and thus their exclusive social nature is not always made clear, allowing for the inaccurate and harmful conclusion that differences observed with such race and ethnicity variables have a biological basis. Textbox 1 offers definitions of different measures related to these 2 specific sensitive variables and highlights how interpretations can be conflated and may not align with their intended use.

**Table 1:** Commonly used fairness metrics [18-21]. 
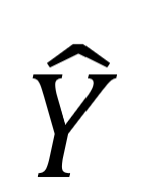
=Prediction or decision, Y=Observed data, and S=Sensitive feature, in the case of S being a multi-group variable where a comparison with a reference or privileged group is meaningful. 
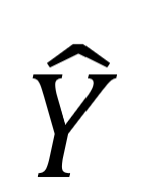
 and Y are binary variables.

Metric	Equation	Definition
Equalized odds	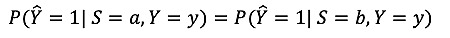	Both groups should have equal true positive and false positive rates.
Equal opportunity	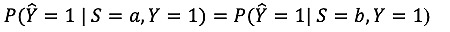	Both groups should have equal true positive rates. A relaxed version of equalized odds.
Predictive equality	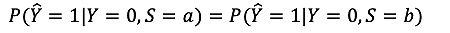	The rate of false positives (negative events categorized as positives) should be independent of the sensitive feature.
False negative rate parity	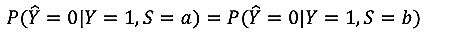	The rate of false negatives (positive events categorized as negatives) should be independent of the sensitive feature.
Predictive parity	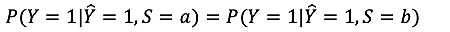	Model precision should be the same for both groups.
Demographic parity	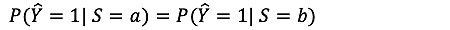	The prediction or decision should be independent of the sensitive feature.

The risk of including sensitive variables only to misinterpret them in a way that furthers inequity has led to an ongoing debate about the place of these sensitive features in clinical risk prediction models. Exclusion of such sensitive features is 1 possible solution, mostly discussed in the context of self-reported race or ethnicity [22], though this kind of “fairness through unawareness” is not always successful and can in some cases further inequities [23]. The use of appropriate fairness metrics is another possible solution. Even when fairness metrics are used, however, the operationalization regarding the measurement of sensitive variables like race or ethnicity can differ from culture to culture, posing further challenges in assessing fairness. For example, while the United States predominantly gathers data on race rather than ethnicity, many European countries focus instead on ethnicity and nationality as indications of socially constructed differences [24]. The use of fairness metrics for sensitive variables that mire diverse definitions across geography and clinical studies remains a complex issue. Sociocultural adaptation of the definition and measurement of sensitive variables is needed for appropriate adoption of fairness. As our focus is on the specific contextual application of fairness metrics, we leave the nuances of this broader issue to many other excellent papers that explore the concept at length [3-5,9,12,18,19,24].

As prediction models are increasingly embedded into electronic health records to realize the goal of precision health [25,26], determining the intended use of sensitive features is crucial. How widely such metrics are used or reported in the clinical risk prediction literature is rarely objectively quantified. Thus, to gather empirical evidence to substantiate our viewpoint, we sought to examine the usage of fairness metrics in clinical risk prediction through a scoping review of recently published risk prediction models in high-impact journals for two diseases: cardiovascular disease (“CVD,” a long-studied chronic and noncommunicable disease) and COVID-19 (a recently emerged infectious disease). Before looking at the data, we hypothesized that there would be little reporting of fairness metrics in CVD research, where many studies span years and began long before discussions of fairness metrics in the prediction literature, but that the emergence of the COVID-19 pandemic would pose an opportunity to more frequently incorporate modern advances in fairness metrics into predicting disease outcomes.

## Methods

A scoping literature review was conducted for each disease of interest. Our outcomes of interest differed slightly between CVD and COVID-19: the clinical risk prediction models for CVD focused on the fatal and nonfatal risks of CVD, while the models for the COVID-19 pandemic focused on the risk of mortality and severe disease. We did not use a classic systematic literature review approach, as our priority was to expediently capture the trend in the highest impact papers. We sought only high-impact papers to understand the prevailing, dominant practices of the field. Figure S1 in Multimedia Appendix 1 details the reproducible steps taken and search terms used for data collection and extraction.

The criteria for highly impactful publications differed between CVD and COVID-19, reflecting the difference between a long-studied disease and an emerging area of study. CVD papers from the last 10 years (2013-2023) were reviewed and selected if they exceeded 100 citations and were from journals with impact factors exceeding 5, while COVID-19 papers from 2021 to 2023 were reviewed and selected if they exceeded 50 citations for 2021 papers or exceeded 10 citations for 2022 to 2023 papers, both from journals with impact factors over 5. Systematic reviews and meta-analysis papers were excluded from the results.

## Results

### Results From the Literature Review on CVD

**Figure 1:**
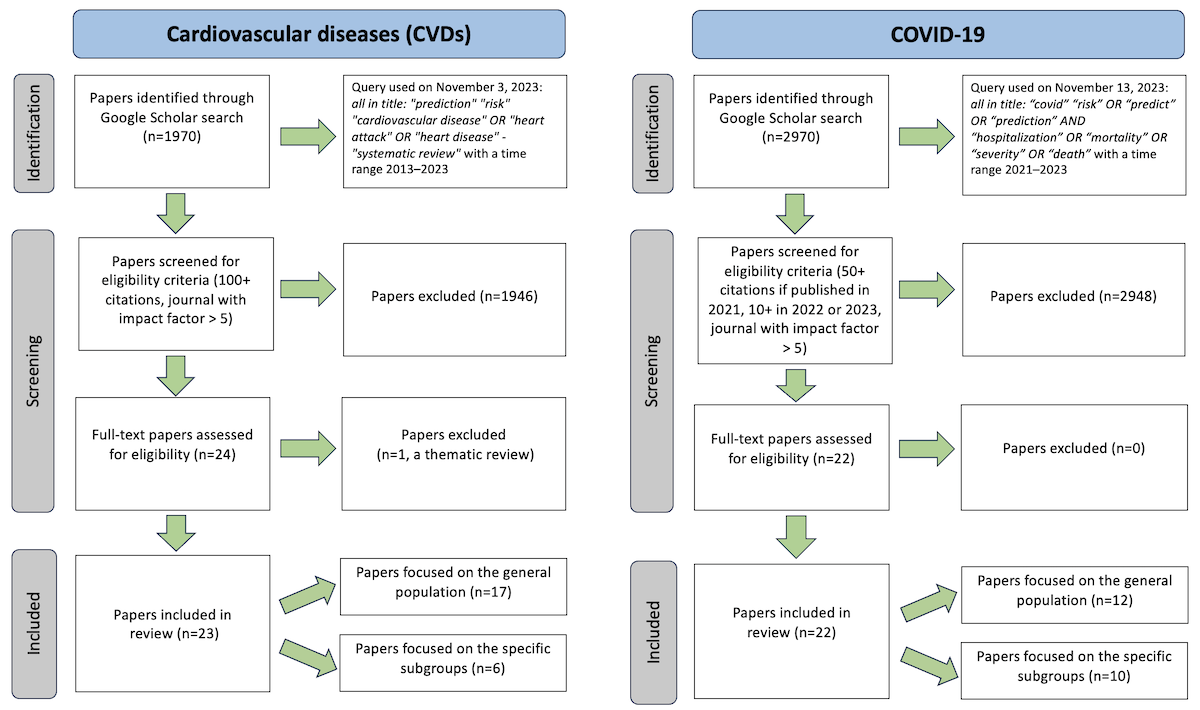
PRISMA flow diagram of the literature search process.

The Google Scholar search query: allintitle: “prediction” “risk” “cardiovascular disease” OR “heart attack” OR “heart disease” OR “mortality” OR “death” -“systematic review” with a time range of 2013-2023 on November 3, 2023, returned 1970 results (Figure 1). For the purposes of our informal review, we reviewed the top 1000 returned by the Google Scholar algorithm. The yielded results were selected if they exceeded 100 citations and were from journals with impact factors exceeding 5. This provided a shortlist of 23 articles predicting the risk of a fatal or nonfatal CVD event that we then divided based on the target population (general population or a specific subpopulation). Tables S1 and S2 in Multimedia Appendix 2 and Figure S1 in Multimedia Appendix 1 of the supplementary material provide details.

**Figure 2:**
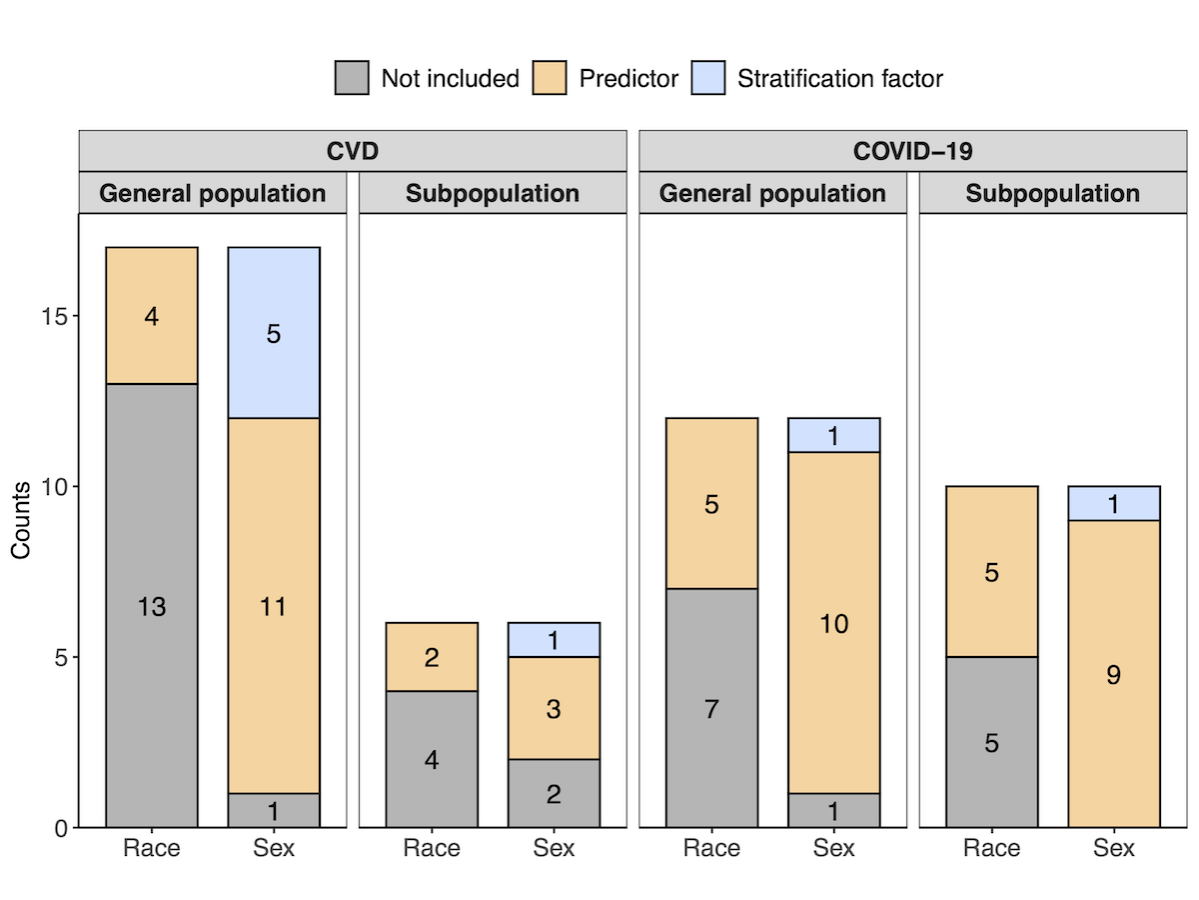
Counts depicting how many of the reviewed articles include race or ethnicity and sex as either predictors or stratification factors in their clinical risk prediction models, separated by disease of interest and population focus of the article. For cardiovascular diseases (CVD), the outcome considered was the risk of cardiovascular disease, heart attack, or heart failure, whereas for the COVID-19 pandemic, it was hospitalization and death.

Of the 17 CVD papers focusing on a general population that met the criteria of this review (Table S1 of Multimedia Appendix 2), none discussed fairness metrics. Of these 17 papers, 5 (29%) stratified their models by sex (ie, built different models for each sex), and 11 (65%) included sex as a covariate. Of these 17 papers, 9 (53%) included data on race or ethnicity (as many of the papers paired race and ethnicity together or used them interchangeably, we will refer to any discussion of either as race or ethnicity jointly, despite this being an imprecise practice). Only 5 of these 9 were multiracial or multiethnic (more than one racial or ethnic group identified), and 4 of these studies included race or ethnicity as a covariate. No study stratified its model by race or ethnicity (Figure 2). The 8 studies that did not include race or ethnicity data were based in the United States or Europe. Other sensitive features considered in the studies include body mass index, stratification by CVD mortality risk regions, and area-based measures of deprivation.

Similar results were observed in the 6 CVD papers that focused on specific subpopulations, ranging from those with chronic conditions to those from specific ethnic or age groups (Table S2 in Multimedia Appendix 2). Of these 6 studies, 3 (50%) included sex as a covariate; only 1 stratified the prediction model by sex (Figure 2). All but one study included self-reported race or ethnicity data, yet only 3 (50%) were multiracial or multiethnic, and only 2 (33%) considered race or ethnicity as a covariate. Other sensitive features considered in the studies include geographic region, level of urbanization, and obesity.

Considering the general population and subpopulation papers together, 6 of the 23 (26%) CVD papers used a sex-stratified model, and of the 14 papers with race or ethnicity data, 13 (92%) had study populations where over 50% of participants were of a particular race or ethnicity.

### Results From the Literature Review on COVID-19

The Google Scholar search query: allintitle: “covid” “risk” “predict” OR “prediction” AND “hospitalization” OR “mortality” OR “severity” OR “death” with a time range of 2021-2023 on November 13, 2023, returned 2970 results. For the purposes of our informal review, we reviewed the top 1000 returned by the Google Scholar algorithm. These results were then selected if they exceeded 50 citations for 2021 papers and exceeded 10 citations for 2022 to 2023 papers, both from journals with impact factors over 5. This yielded a shortlist of 22 articles predicting the risk of COVID-19 hospitalization or death that we then divided based on the target population (general population or a specific subpopulation). Tables S3 and S4 in Multimedia Appendix 2 and Figure S1 in Multimedia Appendix 1 provide details.

Of the 12 COVID-19 papers (Table S3 in Multimedia Appendix 2) focusing on a general population that met the inclusion criteria, none mention fairness metrics. Of the 12 papers, 10 (83.3%) have sex as a covariate included in the prediction model; only one (8.3%) paper stratified participants by sex. These 5 of the 12 (41.7%) papers considered race or ethnicity, all of which included it as a covariate; none stratified by race or ethnicity (Figure 2). Of the remaining 7 papers, 3 cited a dearth of diverse data as the reason for lacking race or ethnicity information; 1 assumed the population to be entirely White; and the other 3 made no comments on race or ethnicity. Other sensitive covariates explored include patient-level socioeconomic index, geographical region, or hospital region.

Similar results were observed in the 10 COVID-19 papers that focused on specific subpopulations, ranging from those with pre-existing chronic diseases to age-specific groups like older adults and infants (Table S4 in Multimedia Appendix 2). All 10 papers included age as a risk factor. Similarly, all 10 considered sex in their models. One paper implemented stratification by sex, while all others (90%) used sex as a covariate. Of these 10 papers, 5 (50%) included race or ethnicity data, and all used it as a covariate. Though there was very little stratification by sex and none by race or ethnicity (Figure 2), stratification was undertaken for various other risk factors, such as the type of medication used and geographical location.

Considering the general population and subpopulation papers together, 2 of the 22 COVID-19 papers (9%) used a sex-stratified model, and of the 10 papers with race or ethnicity data, 5 (50%) had study populations where over 50% of participants were of a particular race or ethnicity.

## Discussion

In our literature review, we found that assessing differences in model performance across sensitive features was somewhat common (eg, comparing areas under the receiver operating characteristic curves across sex or race), but discussing the implications of these differences for fairness and using appropriate fairness metrics were rare. There are several limitations of our approach. Our search was limited to highly cited papers in journals with high impact factors using the Google Scholar database and could have missed important articles; furthermore, we focused on only 2 diseases out of the many for which clinical risk prediction models are developed, restricting the generalizability of our findings to the broader class of clinical risk prediction models. Even though COVID-19 modeling began well after the field of fairness metrics was flourishing in quantitative research, in our review, their usage was as absent among COVID-19 studies as among CVD studies. It is our view that there are 2 primary reasons for the scarcity of fairness considerations in clinical risk prediction: a lack of diverse data and a lack of clarity on the best practice for using fairness metrics.

A lack of diverse data pervaded the studies in our review and was particularly prominent in terms of variation in race or ethnicity and geography. Though over half of the studies in the identified high-impact CVD-related papers reported on multiracial or multiethnic groups (as in, more than one racial or ethnic group reported) the data used in model development were still predominantly from 1 race or ethnicity. For example, in a study by Hippisley-Cox et al [27] with multiethnic data, though over 9 million patient records were used in the analysis, over 85% of subjects either self-reported their ethnic origin as White or did not report this information; other ethnic origins (like Indian, Black Caribbean, and other groups) made up less than 15% of patients. There was more racially or ethnically diverse data in the COVID-19–related papers: of the 10 papers with multiracial or multiethnic data, only half reported a study population of over 50% 1 race or ethnicity. The observed dominance across a single race or ethnicity implies that, for many studies, the lack of sufficient data to test-train models in each race-ethnic group separately makes fairness assessment challenging to begin with. In terms of geography, there was little diversity (Figure S2 in Multimedia Appendix 3) for either disease: the vast majority of reviewed studies were from the Global North.

Another limitation regarding the diversity of the data involved the definitions of race or ethnicity used in these studies. As mentioned in the Introduction, one important first step in using sensitive variables in risk prediction models is to clearly describe their intended use, such as whether they are designed to capture social attributes, biological features, or lived experiences. Of the 24 papers that had information on race or ethnicity, only 6 explicitly stated that the race or ethnicity variables were self-reported (25%); 2 papers directly described their study populations by genetic ancestry instead of by self-reported race or ethnicity (8.3%). The other 16 papers gave no contextualization to the way race or ethnicity data was measured, interpreted, and used, raising the risk of confusing the social as biological [23]. More exact details on the racial or ethnic makeup of the reviewed papers and how race or ethnicity was used and defined in each study are provided in Tables S1-S4 in Multimedia Appendix 2.

For some studies, however, such as those whose models were stratified by sex or study region, sufficient data did exist for computing and providing fairness metrics. Some of these studies did use discrimination and calibration metrics to assess the overall model fit across different subgroups (such as across race or ethnicity), though such analysis was not routinely conducted. Regardless, high discrimination and calibration alone do not guarantee model fairness, as aggregated data can easily mask inequalities [28,29]. From these studies, it appears that fairness analyses simply have not yet been adopted as a part of the model assessment routine.

The lack of clarity as to how best to approach model fairness may have contributed to this practice. Even when the opportunity for the use of fairness metrics is obvious, the question of how to properly leverage them remains complex. There is no “one size fits all” method or universal fairness metric: instead, the specific context of the target outcome (whether it is a preventative intervention or a limited-resource treatment, for example) must inform how relevant concerns of fairness are, and what metrics can address the concerns [12]. Many fairness criteria are in fact mutually incompatible in practical settings (for example, demographic parity and equalized odds) [5], requiring a case-by-case decision on what kind of fairness (and thus fairness metric) will be most meaningful for the data and situation at hand. The limitations of many of the most common fairness metrics can also make their use inapplicable or unappealing for certain models. For example, most metrics are designed to assess only dichotomous outcomes and would require recalculation if a model’s predictions involved different cutoffs of the underlying continuous measures for different decisions. Yet, no critical number of developed fairness metrics will change practice; it is not an issue of lack of methods, but of implementation. Despite similar challenges, fairness methods have already been adopted in a variety of other predictive modeling fields, including criminal justice, finance, and computational linguistics [30-32].

One may pose the question if any clinical risk prediction modelers have used fairness metrics successfully but did not qualify for our scoping review due to its focus on only CVD and the COVID-19 pandemic and only highly cited papers in high-impact journals. To answer this question, we ran a high-level PubMed query of “fairness” AND “risk prediction” in all fields on January 6, 2025. This search returned 5 papers [33-37] on newly developed clinical risk prediction models for a variety of diseases, including preeclampsia, postpartum depression, and CVD, one of our diseases of interest. These papers made fairness metrics a core part of their model assessment and diagnostics but did not meet our criteria for inclusion in our review. For example, Liu et al [33] postpartum depression prediction model was first made to satisfy equal error rates between White and non-White individuals as the primary fairness metric; then debiasing techniques, including reweighing and fairness through blindness, were tested to improve the positive prediction rates between White and non-White individuals. These 5 studies acknowledged the limitations of fairness metrics and the impossibility of satisfying them all simultaneously and clearly defined their sensitive attributes. These new papers and others [38-40] can serve as helpful reference points for researchers as examples of attainable initial starts for fairness considerations when sensitive variables are measured.

**Figure 3:**
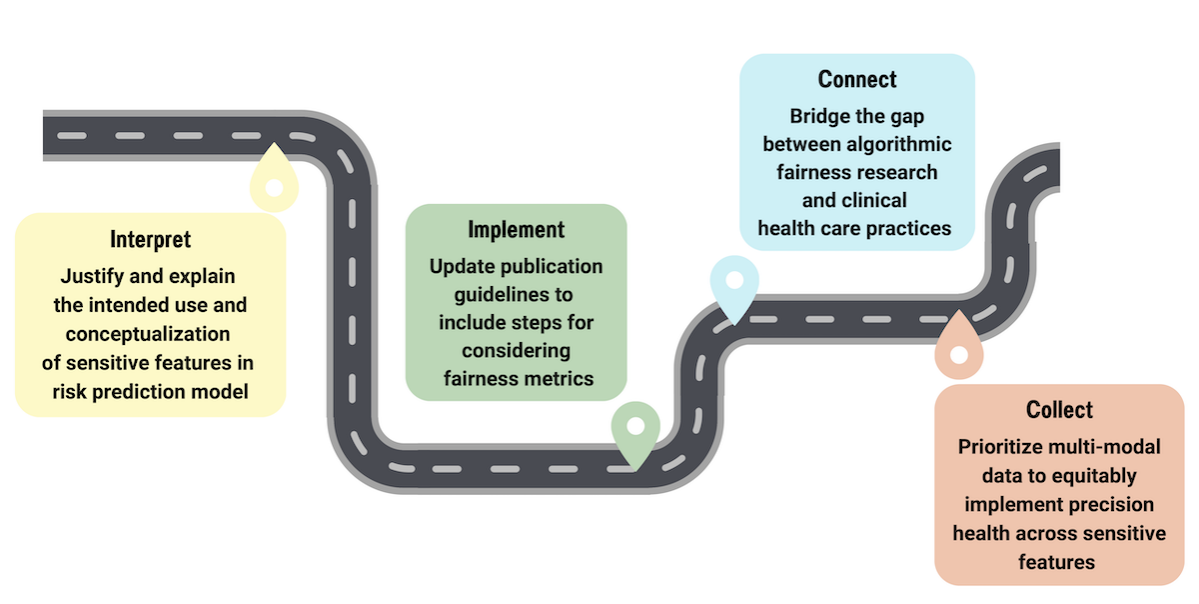
Strategies for increasing the adoption of fairness of clinical risk prediction models in precision health: Interpret, Implement, Connect and Collect (I2C2).

Our own recommendations for how one can permeate and change practice in terms of adopting fairness as a core criterion and principle in clinical risk prediction are listed below and illustrated in the roadmap of Figure 3.

### Roadmap and Recommendations for Changing Practice

We recommend the following 4 strategies for increasing the fairness of clinical risk prediction models across sensitive variables in precision health practice (I2C2).

- **Interpret**: In line with the NIH’s requirement of including (or justifying the exclusion of) a biological sex variable [41], papers should interpret, justify, and explain their intended use and conceptualization of sensitive features in risk prediction models.
- **Implement**: Influential guidelines like EQUATOR (Enhancing the Quality and Transparency of Health Research) TRIPOD (Transparent Reporting of a multivariable prediction model for Individual Prognosis or Diagnosis) guidelines [42] should include steps on considering algorithmic fairness as part of the implementation and application of clinical risk prediction models.
- **Connect**: The community of methods research in algorithmic fairness should ensure that the methods and tools developed are well broadcast to those in the community of practice in clinical health care, connecting theory to practice.
- **Collect**: The fields of clinical risk prediction and precision health should highly prioritize collecting inclusive, multimodel data across race, geographic region, and a variety of other sensitive or historically underrepresented features.

To understand the current barriers in the precision health practice community, we have developed a short questionnaire to help authors identify key challenges in implementing fairness metrics. This questionnaire will help clarify where resources should be most concentrated for this I2C2 roadmap. The questionnaire can be accessed here, and Table S5 in Multimedia Appendix 4 shows the summary questions. We hope by educating and enabling public health and clinical practitioners on the importance of assessing fairness metrics, we will create a more equitable prediction and precision prevention world for all.

All materials used for this review can be accessed via the GitHub of the University of Michigan Center for Precision Health Data Science.

## Data Availability

All materials used for this review can be accessed via the GitHub of the University of Michigan Center for Precision Health Data Science.

## Appendix

Multimedia Appendix 1 PRISMA checklist.

Multimedia Appendix 2 Articles selected for review. Includes Tables S1-S4.

Multimedia Appendix 3 Counts depicting the geographic origin of data used in the reviewed articles, separated by disease of interest and population focus of the article. Studies with study regions covering multiple continents are double counted.

Multimedia Appendix 4 The ten questions included in the questionnaire. All but questions 9 and 10 are multiple choice, with the option to elaborate in a free response. Supplementary Table 5.

## Abbreviations

CVD: cardiovascular disease
EQUATOR: Enhancing the Quality and Transparency of Health Research
PRISMA: Preferred Reporting Items for Systematic Reviews and Meta-Analyses
TRIPOD: Transparent Reporting of a multivariable prediction model for Individual Prognosis or Diagnosis

## References

[1] Framingham Heart Study FHS Cardiovascular disease (10-year risk). Framingham Heart Study.

[2] Breast cancer risk assessment tool: online calculator (the Gail Model). National Cancer Institute.

[3] Bærøe K, Gundersen T, Henden E, Rommetveit K (2022). Can medical algorithms be fair? Three ethical quandaries and one dilemma. BMJ Health & Care Informatics.

[4] Mitchell S, Potash E, Barocas S, D'Amour A, Lum K (2021). Algorithmic fairness: choices, assumptions, and definitions. Annu. Rev. Stat. Appl.

[5] Mehrabi N, Morstatter F, Saxena N, Lerman K, Galstyan A (2021). A survey on bias and fairness in machine learning. ACM Comput. Surv.

[6] Wiens J, Spector-Bagdady K, Mukherjee B (2024). Toward realizing the promise of AI in precision health across the spectrum of care. Annual Review of Genomics and Human Genetics.

[7] Biden JR Executive Order on the Safe, Secure, and Trustworthy Development and Use of Artificial Intelligence. Federal Register.

[8] Coiera E, Fraile-Navarro D (2024). AI as an Ecosystem — Ensuring Generative AI Is Safe and Effective. NEJM AI.

[9] Paulus JK, Kent DM (2017). Race and Ethnicity: A Part of the Equation for Personalized Clinical Decision Making?. Circulation: Cardiovascular Quality and Outcomes.

[10] Lu C, Ahmed R, Lamri A, Anand SS (2022). Use of race, ethnicity, and ancestry data in health research. PLOS Glob Public Health.

[11] Tannenbaum C, Ellis RP, Eyssel F, Zou J, Schiebinger L (2019). Sex and gender analysis improves science and engineering. Nature.

[12] Paulus JK, Kent DM (2020). Predictably unequal: understanding and addressing concerns that algorithmic clinical prediction may increase health disparities. NPJ Digit Med.

[13] Fletcher RR, Nakeshimana A, Olubeko O (2020). Addressing Fairness, Bias, and Appropriate Use of Artificial Intelligence and Machine Learning in Global Health. Front Artif Intell.

[14] Parbhoo S, Wawira Gichoya J, Celi LA, de la Hoz MA, for MIT Critical Data (2022). Operationalising fairness in medical algorithms. BMJ Health Care Inform.

[15] Adebayo JA (2016). Massachusetts institute of technology. FairML: ToolBox for diagnosing bias in predictive modeling.

[16] Kozodoi N, Varga TV (2020). Algorithmic Fairness in R. Nikita Kozodoi.

[17] Nabi R, Shpitser I (2018). Fair inference on outcomes. AAAI.

[18] Castelnovo A, Crupi R, Greco G, Regoli D, Penco IG, Cosentini AC (2022). A clarification of the nuances in the fairness metrics landscape. Sci Rep.

[19] Verma Sahil, Rubin Julia (2018). Fairness Definitions Explained. Association for Computing Machinery.

[20] Chouldechova A (2017). Fair prediction with disparate impact: a study of bias in recidivism prediction instruments. Big Data.

[21] Hardt M, Price E, Srebro N (2016). Equality of Opportunity in Supervised Learning. arXiv.

[22] Vyas DA, Eisenstein LG, Jones DS (2020). Hidden in Plain Sight - Reconsidering the Use of Race Correction in Clinical Algorithms. N Engl J Med.

[23] Basu A (2024). Mitigating Clinical Algorithmic Discrimination. JAMA Health Forum.

[24] Jaime Sofia, Kern Christoph (2024). Ethnic classifications in algorithmic fairness: concepts, measures, and implications in practice.

[25] Sharma V, Ali I, van der Veer S, Martin G, Ainsworth J, Augustine T (2021). Adoption of clinical risk prediction tools is limited by a lack of integration with electronic health records. BMJ Health Care Inform.

[26] Lee TC, Shah NU, Haack A, Baxter SL (2020). Clinical Implementation of Predictive Models Embedded within Electronic Health Record Systems: A Systematic Review. Informatics (MDPI).

[27] Hippisley-Cox J, Coupland CAC, Mehta N, Keogh RH, Diaz-Ordaz K, Khunti K, Lyons RA, Kee F, Sheikh A, Rahman S, Valabhji J, Harrison EM, Sellen P, Haq N, Semple MG, Johnson PWM, Hayward A, Nguyen-Van-Tam JS (2021). Risk prediction of covid-19 related death and hospital admission in adults after covid-19 vaccination: national prospective cohort study. BMJ.

[28] Loi M, Heitz C (2022). Is calibration a fairness requirement? An argument from the point of view of moral philosophy and decision theory.

[29] Kauh TJ, Read JG, Scheitler AJ (2021). The Critical Role of Racial/Ethnic Data Disaggregation for Health Equity. Popul Res Policy Rev.

[30] Wang C, Han B, Patel B, Rudin C (2022). In Pursuit of Interpretable, Fair and Accurate Machine Learning for Criminal Recidivism Prediction. J Quant Criminol.

[31] Te YF, Wieland M, Frey M, Grabner H (2023). Mitigating discriminatory biases in success prediction models for venture capitals.

[32] Schwertmann L, Kannan Ravi MP, de Melo G (2023). Model-Agnostic Bias Measurement in Link Prediction. Findings of the Association for Computational Linguistics: EACL 2023.

[33] Liu Y, Joly R, Reading Turchioe M, Benda N, Hermann A, Beecy A, Pathak J, Zhang Y (2024). Preparing for the bedside-optimizing a postpartum depression risk prediction model for clinical implementation in a health system. J Am Med Inform Assoc.

[34] Atehortúa A, Gkontra P, Camacho M, Diaz O, Bulgheroni M, Simonetti V, Chadeau-Hyam M, Felix JF, Sebert S, Lekadir K (2023). Cardiometabolic risk estimation using exposome data and machine learning. Int J Med Inform.

[35] Topaz M, Davoudi A, Evans L, Sridharan S, Song J, Chae S, Barrón Y, Hobensack M, Scharp D, Cato K, Rossetti S, Kapela P, Xu Z, Gupta P, Zhang Z, Mcdonald M, Bowles K (2025). Building a time-series model to predict hospitalization risks in home health care: insights into development, accuracy, and fairness. J Am Med Dir Assoc.

[36] Li C, Jiang X, Zhang K (2024). A transformer-based deep learning approach for fairly predicting post-liver transplant risk factors. J Biomed Inform.

[37] Lin YC, Mallia D, Clark-Sevilla AO, Catto A, Leshchenko A, Yan Q, Haas DM, Wapner R, Pe'er Itsik, Raja A, Salleb-Aouissi A (2024). A comprehensive and bias-free machine learning approach for risk prediction of preeclampsia with severe features in a nulliparous study cohort. BMC Pregnancy Childbirth.

[38] Kartoun U, Khurshid S, Kwon BC, Patel AP, Batra P, Philippakis A, Khera AV, Ellinor PT, Lubitz SA, Ng K (2022). Prediction performance and fairness heterogeneity in cardiovascular risk models. Sci Rep.

[39] Foryciarz A, Pfohl SR, Patel B, Shah N (2022). Evaluating algorithmic fairness in the presence of clinical guidelines: the case of atherosclerotic cardiovascular disease risk estimation. BMJ Health Care Inform.

[40] Wastvedt S, Huling JD, Wolfson J (2024). An intersectional framework for counterfactual fairness in risk prediction. Biostatistics.

[41] Arnegard ME, Whitten LA, Hunter C, Clayton JA (2020). Sex as a biological variable: a 5-year progress report and call to action. J Womens Health (Larchmt).

[42] Collins GS, Reitsma JB, Altman DG, Moons KGM (2015). Transparent Reporting of a multivariable prediction model for Individual Prognosis Or Diagnosis (TRIPOD). Ann Intern Med.

