# Supplementary material for: Reporting of Fairness Metrics in Clinical Risk Prediction Models Used for Precision Health: Scoping Review
Source: Online J Public Health Inform. 2025 Mar 19;17:e66598. doi: 10.2196/66598 (PMC11966066; doi:10.2196/66598)
Supplement: Multimedia Appendix 2 [file ojphi_v17i1e66598_app2.pdf]

Appendix

S.1 Table of Selected CVD Papers for General Population

Title	Authors	Link/DOI	Citations	Journal	Year	Outcome assessed	Type of model used	Geographic region	Racial/ethnic demographics	Considered single race/ethnicity or multiracial/multiethnic?	Sensitive features considered as risk factors	Sensitive features considered for stratified analysis	Were model calibration and discrimination assessed for different sensitive features?	Criteria for model evaluation	Do authors explicitly consider or report fairness metrics?
Development and validation of QRISK3 risk prediction algorithms to estimate future risk of cardiovascular disease: prospective cohort study	Hippisley-Cox et al	10.1136/bmj.j2099	923	BMJ	2017	Risk of CVD	Cox proportional hazards models	United Kingdom	Self-reported in ~60% of all subjects. Majority (>85%) White or not recorded. ~2% Indian. ~1% Pakistani. ~1% Bangladeshi. ~1.3% Other Asian. ~1% Black Caribbean. ~2% Black African. ~0.8% Chinese. 2.5% Other.	Multiracial/multiethnic	Age, ethnic origin	Sex	Yes	Harrell's C-statistics	No
Risk prediction of cardiovascular death based on the QTc interval: evaluating age and gender differences in a large primary care population	Nielsen et al	10.1093/eurheartj/ehu081	123	European Heart Journal	2014	Risk of CVD	Cox regression models	Denmark	Not included	N/A	Age	Sex	Yes	C-statistics, Brier scores	No
Cardiovascular disease risk prediction equations in 400 000 primary care patients in New Zealand: a derivation and validation study	Pylypchuk et al	10.1016/S0140-6736(18)30664-0	224	The Lancet	2018	Risk of fatal or non- fatal CVD event	Cox proportional hazards models	New Zealand	Majority (>55%) European. ~13% Maori. ~13% Pacific. ~9% Indian. ~11% Chinese or other Asian (self-reported)	Multiracial/multiethnic	Measures of deprivation, ethnicity, age	Sex	Yes	R^2, Harrell's C statistic, and Royston's D statistic	No
The ACC/AHA 2013 pooled cohort equations compared to a Korean Risk Prediction Model for atherosclerotic cardiovascular disease	Jung et al	10.1016/j.atherosclerosis.2015. 07.033	111	Atherosclerosis	2015	Ten-year atherosclerotic cardiovascular disease risk	Cox proportional hazards models	South Korea	Korean	Single race/ethnicity	Age	Sex	Yes	C-statistic, Hosmer-Lemeshow	No
SCORE2 risk prediction algorithms: new models to estimate 10-year risk of cardiovascular disease in Europe	SCORE2 working group and ESC Cardiovascular risk collaboration	10.1093/eurheartj/ehab309	397	European Heart Journal	2021	Ten-year risk of fatal and non-fatal CVD	Fine and Gray model stratified by cohort and fitted to sex	Europe and North America	Not included	N/A	Age	Risk region, sex	Yes	Harrell's C-Index	No
Risk prediction by genetic risk scores for coronary heart disease is independent of self-reported family history	Tada et al	10.1093/eurheartj/ehv462	276	European Heart Journal	2016	Time to first occurrence of CHD	Cox proportional hazards models	Sweden	Swedish ancestry (genetic)	Single race/ethnicity	Sex, age	N/A	No	Wald tests	No
A Validated Model for Sudden Cardiac Death Risk Prediction in Pediatric Hypertrophic Cardiomyopathy	Miron et al	10.1161/CIRCULATIONAHA.120.047235	121	Circulation	2020	Risk of sudden cardiac death	Cause-specific hazard regression model	Canada, United States, Australia	Not included	N/A	Sex, age	N/A	No	C-statistic, cross validation	No
Multilocus Genetic Risk Scores for Coronary Heart Disease Prediction	Ganna et al	10.1161/ATVBAHA.113.301218	174	Arteriosclerosis, Thrombosis, and Vascular Biology	2013	Risk of coronary heart disease	Cox proportional hazards model	Sweden	Not included	N/A	Sex, age	N/A	No	C-index	No
Improving the accuracy of prediction of heart disease risk based on ensemble classification techniques	Latha and Jeeva	10.1016/j.imu.2019.100203	435	Informatics in Medicine Unlocked	2019	Risk of heart disease	Comparative analysis of various classification ML algorithms	United States	Not included	N/A	Sex, age	N/A	No	Ten-fold cross validation	No

Title	Authors	Link/DOI	Citations	Journal	Year	Outcome assessed	Type of model used	Geographic region	Racial/ethnic demographics	Considered single race/ethnicity or multiracial/multiethnic?	Sensitive features considered as risk factors	Sensitive features considered for stratified analysis	Were model calibration and discrimination assessed for different sensitive features?	Criteria for model evaluation	Do authors explicitly consider or report fairness metrics?
Comparison of machine learning algorithms for clinical event prediction (risk of coronary heart disease)	Beunza et al	10.1016/j.jbi.2019.103257	142	Journal of Biomedical Informatics	2019	Coronary risk at ten years	Machine learning classification algorithms	United States	Not included	N/A	Sex, age	N/A	No	AUC	No
An improved ensemble learning approach for the prediction of heart disease risk	Mienye et al	10.1016/j.imu.2020.100402	136	Informatics in Medicine Unlocked	2020	Risk of CVD event	Ensemble learning	United States	Not included	N/A	Sex, age	N/A	No	ROC	No
Validation of the 2014 European Society of Cardiology Guidelines Risk Prediction Model for the Primary Prevention of Sudden Cardiac Death in Hypertrophic Cardiomyopathy	Vriesendorp et al	10.1161/CIRCEP.114.002553	143	Circulation: Arrhythmia and Electrophysiology	2015	Five-year risk of sudden cardiac death	Cox regression models	Belgium and the Netherlands	Not included	N/A	Age	N/A	No	ROC, C-statistics	No
Endothelial Dysfunction, Increased Arterial Stiffness, and Cardiovascular Risk Prediction in Patients With Coronary Artery Disease: FMD-J (Flow-Mediated Dilation Japan) Study A	Maruhashi et al	10.61/JAHA.118.008588	106	Journal of the American Heart Association	2018	Risk of recurrent cardiovascular events	Cox proportional hazard models	Japan	Japanese	Single race/ethnicity	Sex, age	N/A	No	Schoenfeld residuals	No
Electrical risk score beyond the left ventricular ejection fraction: prediction of sudden cardiac death in the Oregon Sudden Unexpected Death Study and the Atherosclerosis Risk in Communities Study	Aro et al	10.1093/eurheartj/ehx331	115	European Heart Journal	2017	Risk of sudden cardiac arrest	Multivariable logistic regression analysis	United States	In cases: 82% white, 11% Black, 1.9% Hispanic, 5.2% other.<br><br>In controls: 92% white, 3.5% Black, 1.4% Hispanic, 3.3% other.	Multiracial/multiethnic	Sex, age	N/A	No	Hosmer-Lemeshow, C-statistic	No
Genetic Risk Prediction and a 2- Stage Risk Screening Strategy for Coronary Heart Disease	Tikkanen et al	10.1161/ATVBAHA.112.301120	206	Arteriosclerosis, Thrombosis, and Vascular Biology	2013	Fatal and non-fatal CHD risk	Cox regression models	Finland	Finnish ancestry (genetic)	Single race/ethnicity	Sex, age	N/A	No	C-index	No
10-Year Coronary Heart Disease Risk Prediction Using Coronary Artery Calcium and Traditional Risk Factors: Derivation in the MESA (Multi-Ethnic Study of Atherosclerosis) With Validation in the HNR (Heinz Nixdorf Recall) Study and the DHS (Dallas Heart Study)	McClelland et al	10.1016/j.jacc.2015.08.035	561	Journal of the American College of Cardiology	2015	Incident CHD events	Penalized Cox proportional hazards model	United States	A combination of several datasets. One was 100% Caucasian. The largest was 38.5% Caucasian, 11.8% Chinese American, 27.8% African American, 22% Hispanic American. The other was 37.9% Caucasian, 49.1% African American, 11.3% Hispanic American, 1.8% Other.	Multiracial/multiethnic	Age, sex, BMI, race/ethnicity	N/A	Yes	C-statistic and ROC	No
Global Electric Heterogeneity Risk Score for Prediction of Sudden Cardiac Death in the General Population	Waks et al	10.1161/CIRCULATIONAHA.116.021306	131	Circulation: Arrhythmia and Electrophysiology	2016	Risk of sudden cardiac death	Cox proportional hazards models	United States	77.3% white; 22.7% Black (other races excluded)	Multiracial/multiethnic	Sex, race, age	N/A	No	Schoenfeld residuals, C-statistics	No

S.2 Table of Selected CVD Papers for Subpopulations

Title	Authors	Link/DOI	Citations	Journal	Year	Outcome assessed	Type of model used	Geographic region	Racial/ethnic demographics	Considered single race/ethnicity or multiracial/multiethnic?	Sensitive features considered as risk factors	Sensitive features considered for stratified analysis	Were model calibration and discrimination assessed for different sensitive features?	Criteria for model evaluation	Do authors explicitly consider or report fairness metrics?
Predicting the 10-Year Risks of Atherosclerotic Cardiovascular Disease in Chinese Population	Yang et al	10.1161/CIRCULATIONAHA.116.022367	402	Circulation	2016	Ten year risk of fatal and non-fatal CVD	Cox proportional hazards models	China	Chinese	Single race/ethnicity	Age, geographic region (Northern/Southern China), urbanization	Sex	Yes	C-statistics and modified Nam-D'Agostino test	No
Cardiovascular Disease Risk Prediction in the HIV Outpatient Study	Thompson-Paul et al	10.1093/cid/ciw615	146	Clinical Infectious Diseases	2016	Risk of CVD event	Adaptations of Framingham, PCEs, SCORE and DAD	United States	Majority (>50%) White, non- Hispanic. ~30% Black, non-Hispanic. ~12% Hispanic. ~3% Other.	Multiracial/multiethnic	Sex, age	N/A	No	C-statistic and Hosmer-Lemeshow	No
An updated prediction model of the global risk of cardiovascular disease in HIV-positive persons: The Data-collection on Adverse Effects of Anti-HIV Drugs (D:A:D) study	Friis-Møller et al	10.1177/2047487315579291	228	European Journal of Preventative Cardiology	2020	Risk of CVD event	Cox regression models	Europe and Australia	Majority (>60%) White. ~7% Non-White. 32% Unknown. (Self-reported)	Multiracial/multiethnic	Age, sex, ethnicity	N/A	No	C-statistic and Hosmer-Lemeshow	No
Development of a Novel Risk Prediction Model for Sudden Cardiac Death in Childhood Hypertrophic Cardiomyopathy (HCM Risk-Kids)	Norrish et al	10.1001/jamacardio.2019.2861	135	JAMA Cardiology	2019	Risk of sudden cardiac death or equivalent event	Cox proportional hazards regression models	Western Europe, Eastern Europe, Japan, Australia	Not included	N/A	None	N/A	No	Schoenfeld residuals, C-index	No
Anthropometric measurements of general and central obesity and the prediction of cardiovascular disease risk in women: a cross-sectional study	Goh et al	10.1136/bmjopen-2013-004138	204	BMJ Open	2014	Ten-year CVD risk	Framingham risk score model, SCORE risk chart for high-risk regions, general CVD and simplified general CVD risk score models	Australia	Reported as ethnicity: Australia (76.5%); UK and Ireland (9.5%); Northern Europe (4.1%); Southern Europe (5.4%); Asia (4.5%).	Multiracial/multiethnic	Obesity, ethnicity	N/A	No	ROC	No
Prediction of First Cardiovascular Disease Event in Type 1 Diabetes Mellitus	Vitisen et al	10.1161/CIRCULATIONAHA.115.018844	104	Circulation	2016	Fatal and non-fatal CVD risk	Poisson regression analysis	Denmark	White (>90% Danish ancestry)	Single race/ethnicity	Sex, age	N/A	No	Hosmer-Lemeshow	No

S.3 Table of Selected COVID-19 Papers for General Population

Title	Authors	Link/DOI	Citations	Journal	Year	Outcome assessed	Type of model used	Geographic region	Racial/ethnic demographics	Considered single race/ethnicity or multiracial/multiethnic?	Sensitive features considered as risk factors	Sensitive features considered for stratified analysis	Were model calibration and discrimination assessed for different sensitive features?	Criteria for model evaluation	Do authors explicitly consider or report fairness metrics?
Predicting mortality risk in patients with COVID-19 using machine learning to help medical decision- making	Pourhomayoun and Shakibi	10.1016/j.smhl.2020.100178	246	Smart Health	2021	Risk of mortality	Multiple ML methods	146 countries	Not included	N/A	Age, sex, country	N/A	No	Accuracy, sensitivity, specificity, AUC	No
Clinical, radiological, and laboratory characteristics and risk factors for severity and mortality of 289 hospitalized COVID-19 patients	Zhang et al.	10.1111/all.14496	204	Allergy	2021	Risk of in-hospital mortality	Logistic regression	China	Not included	N/A	Age, sex	N/A	No	AUC	No
Clinical and inflammatory features based machine learning model for fatal risk prediction of hospitalized COVID-19 patients: results from a retrospective cohort study	Guan et al.	10.1080/07853890.2020.1868564	109	Annals of Medicine	2021	Risk of mortality	XGBoost	China	Not included	N/A	Age	N/A	No	AUC, prediction accuracy, precision, and F1 scores	No
Risk factors for COVID-19 progression and mortality in hospitalized patients without pre-existing comorbidities	Liu et al.	10.1016/j.jiph.2021.11.012	26	Journal of Infection and Public Health	2022	Risk of mortality	Logistic regression	China	Not included	N/A	Sex, age	N/A	No	Not specified	No
Machine learning approaches in Covid-19 severity risk prediction in Morocco	Laatifi et al.	10.1186/s40537-021-00557-0	27	Journal of Big Data	2022	Risk of severity	UMAP, logistic regression, SVM, KNN, Gaussian NB, decision tree	Morocco	Not included	N/A	Sex, age	N/A	No	Accuracy, sensitivity, specificity, AUC	No
External validation of the QCovid risk prediction algorithm for risk of COVID-19 hospitalisation and mortality in adults: national validation cohort study in Scotland	Simpson et al.	10.1136/thoraxjnl-2021-217580	17	Thorax	2022	Risk of hospitalization, risk of death	QCOVID algorithm (Fine-Gray sub-distribution hazard model)	Scotland, United Kingdom	Not included	N/A	Socioeconomic status	Sex, age	No	Harrell's C, R^2, D-statistics, Brier Score	No
Predicting the evolution of COVID- 19 mortality risk: A recurrent neural network approach	Villegas et al.	10.1016/j.cmpbup.2022.100089	13	Computer Methods and Programs in Biomedicine	2023	Risk of mortality	Recurrent neural network	Spain	Not included	N/A	Sex, age	N/A	No	Accuracy, sensitivity, specificity, AUC	No
Body Mass Index and Risk for COVID-19-Related Hospitalization, Intensive Care Unit Admission, Invasive Mechanical Ventilation, and Death - United States, March-December 2020	Kompaniyets et al.	10.15585/mmwr.mm7010e4	394	Morbidity and Mortality Weekly Report	2021	Risk of hospitalization, risk of ICU admission, risk of death	Multivariable logit model	United States	Hispanic (10.4 %), White non- Hispanic (63.7%), Black non-Hispanic (18.4%), Asian non- Hispanic (2.1%), Other (4%), Unknown (1.4%)	Multiracial/multiethnic	Sex, race	Age, BMI	No	Sensitivity analysis	No

Title	Authors	Link/DOI	Citations	Journal	Year	Outcome assessed	Type of model used	Geographic region	Racial/ethnic demographics	Considered single race/ethnicity or multiracial/multiethnic?	Sensitive features considered as risk factors	Sensitive features considered for stratified analysis	Were model calibration and discrimination assessed for different sensitive features?	Criteria for model evaluation	Do authors explicitly consider or report fairness metrics?
Risk factors for mortality in patients with COVID-19 in New York City	Mikami et al.	10.1007/s11606-020-05983-z	286	Journal of General Internal Medicine	2021	Risk of in-hospital mortality	Generalized additive models and Cox proportional hazard regression model	United States	White (26.9%), Black (24.1%), Asian (4.4%), Others (44.7%), Hispanic (25.4%), Non-Hispanic (57.5%), Unknown ethnicity (17%) (self-reported)	Multiracial/multiethnic	Age, sex, race, ethnicity	N/A	No	AUC	No
Federated learning of electronic health records to improve mortality prediction in hospitalized patients with COVID-19: machine learning approach	Vaid et al.	10.2196/24207	100	JMIR Medical Informatics	2021	Risk of mortality	Multilayer perceptron (MLP) model, logistic regression, federate learning model	United States	Hispanic (26.07%), non-Hispanic (59.45%), Unknown ethnicity (14.48%); White (23.9%), Black (28.54%), Asian (4.84%), Other (38.57%), Unknown (4.14%)	Multiracial/multiethnic	Sex, race, ethnicity	N/A	No	AUC	No
Estimating risk of mechanical ventilation and in-hospital mortality among adult COVID-19 patients admitted to Mass General Brigham: The VICE and DICE scores	Nicholson et al.	10.1016/j.eclinm.2021.100765	83	EClinical Medicine	2021	Ventilation in COVID Estimator [VICE] score and Death in COVID Estimator [DICE] score	Logistic regression	United States	White (42%), Black (17.9%), Hispanic (10.8%), Asian (3.6%), other/mix (17%), not recorded (8.6%)	Multiracial/multiethnic	Weight, age, sex, race	N/A	No	AUC and C-statistics	No
Machine-learning-based COVID-19 mortality prediction model and identification of patients at low and high risk of dying	Banoei et al.	10.1186/s13054-021-03749-5	63	Critical Care	2021	Mortality outcome and clustering of high mortality risk patients	Statistically inspired modification of partial least square (SIMPLS) analysis	United States	European American (63.5%), African American, (15.25%), Asian (1%), More than one race (5%), Hispanic (59.75%), non-Hispanic (40.25%)	Multiracial/multiethnic	Age, sex, race, smoking, mental status	N/A	No	Q^2, R^2, and AUC	No

S.4 Table of Selected COVID-19 Papers for Subpopulation

Title	Authors	Link/DOI	Citations	Journal	Year	Outcome assessed	Type of model used	Geographic region	Racial demographics	Considered single race/ethnicity or multiracial/multiethnic?	Sensitive features considered as risk factors	Sensitive features considered for stratified analysis	Were model calibration and discrimination assessed for different sensitive features?	Criteria for model evaluation	Do authors explicitly consider or report fairness metrics?
Clinical characteristics and risk factors for death among hospitalized children and adolescents with COVID-19 in Brazil: an analysis of a nationwide database	Oliveira et al.	10.1016/S2352-4642(21)00134-6	112	The Lancet Child and Adolescent Health	2021	Time to recovery or time to death	Proportional sub-distribution hazards model	Brazil	White (35.24%), Black or Brown (62.42%), Asian (0.88%), Indigenous (1.44%). (Self-reported)	Multiracial/multiethnic	Age, sex, ethnicity, geopolitical macroregion	N/A	No	Competing risks analysis	No
Association between antidepressant use and reduced risk of intubation or death in hospitalized patients with COVID-19: results from an observational study	Hoertel et al.	10.1038/s41380-021-01021-4	208	Molecular Psychiatry	2021	Time from study baseline to intubation or death	Cox regression proportional hazard models	France	Not included	N/A	Sex, age, obesity, smoking status	N/A	No	Sensitivity analysis	No
Hypertension, diabetes and obesity, major risk factors for death in patients with COVID-19 in Mexico	Peña et al.	10.1016/j.arcmed.2020.12.002	138	Archives of Medical Research	2021	Risk of mortality	Logistic regression	Mexico	Not included	N/A	Age, sex, smoking status	N/A	No	Not specified	No
Clinical characteristics and risk factors for mortality in very old patients hospitalized with COVID-19 in Spain	Ramos-Rincon et al.	10.1093/gerona/glaa243	123	The Journals of Gerontology: Series A	2021	Risk of in-hospital mortality	Logistic regression	Spain	Not included	N/A	Age, sex	N/A	No	Hosmer–Lemeshow test	No
Risk of infection, hospitalization, and death up to 9 months after a second dose of COVID-19 vaccine: a retrospective, total population cohort study in Sweden	Nordström et al.	10.1016/S0140-6736(22)00089-7	209	The Lancet	2022	Risk of infection, risk of severe COVID	Cox proportional hazards models	Sweden	Not included	N/A	Age, sex, born in Sweden or not	N/A	No	Schoenfeld residuals	No
Risk prediction of covid-19 related death and hospital admission in adults after covid-19 vaccination: national prospective cohort study	Hippisley-Cox et al.	10.1136/bmj.n2244	262	BMJ	2021	Time to COVID-19 related death and time to hospitalization	Cause-specific Cox proportional hazard models	United Kingdom	White (68.77%), Indian (2.91%), Pakistani (1.61%), Bangladeshi (1.17%), Other Asian (1.68%), Caribbean (0.7%), Black African (1.63%), Chinese (0.6%), Other (2.7%)	Multiracial/multiethnic	Age, sex, ethnic origin	N/A	No	C-statistics, R squared, D-statistics	No
Comparison of mortality risk in patients with cirrhosis and COVID- 19 compared with patients with cirrhosis alone and COVID-19 alone: multicentre matched cohort	Bajaj et al.	10.1136/gutjnl- 2020-322118	207	Gut	2021	Risk of mortality	Logistic regression	United States	White (56.25%), Non-white (43.75%), Hispanic ethnicity (7.35%), non-Hispanic ethnicity (92.65%)	Multiracial/multiethnic	Sex, age, race and ethnicity, smoking status	N/A	No	Not specified	No

Title	Authors	Link/DOI	Citations	Journal	Year	Outcome assessed	Type of model used	Geographic region	Racial demographics	Considered single race/ethnicity or multiracial/multiethnic?	Sensitive features considered as risk factors	Sensitive features considered for stratified analysis	Were model calibration and discrimination assessed for different sensitive features?	Criteria for model evaluation	Do authors explicitly consider or report fairness metrics?
Metformin and risk of mortalityin patients hospitalised with COVID-19: a retrospective cohort analysis	Bramante et al.	10.1016/S2666-7568(20)30033-7	156	The Lancet Healthy Longevity	2021	Risk of in-hospital mortality	Logistic regression, mixed-effect logistic regression, Cox proportional hazard models, propensity-matched mixed-effects logistic regression	United States	Not included	N/A	Sex	No	Schoenfeld residuals, sensitivity analysis	No	No
Maternal vaccination and risk of hospitalization for Covid-19 among infants	Halasa et al.	10.1056/NEJMoa2204399	112	The New England Journal of Medicine	2022	Risk of hospitalization	Logistic regression	United States	White non-Hispanic (39.18%), Black non-Hispanic (17.54%), Hispanic, any race (28.79%), other non-Hispanic (6.39%), Unknown (8.1%)	Multiracial/multiethnic	Age, sex, race and ethnicity, region	N/A	No	Not specified	No
Diet quality and risk and severity of COVID-19: a prospective cohort study	Merino et al.	10.1136/gutjnl-2021-325353	144	Gut	2021	Risk of infection, risk of severe COVID	Cox regression proportional hazard models	United States and United Kingdom	White (96%), Black (0.7%), Asian (1.8%), Other (1.2%), Missing (0.3%) (Self-reported)	Multiracial/multiethnic	Socio-economic status, smoking, race/ethnicity	No	No	Schoenfeld residuals	No
