# Supplementary figures and images for: Reporting of Fairness Metrics in Clinical Risk Prediction Models Used for Precision Health: Scoping Review
Source: Online J Public Health Inform. 2025 Mar 19;17:e66598. doi: 10.2196/66598 (PMC11966066; doi:10.2196/66598)

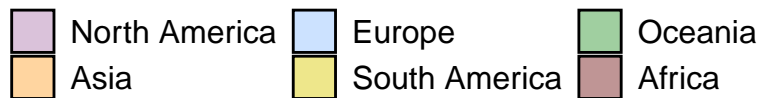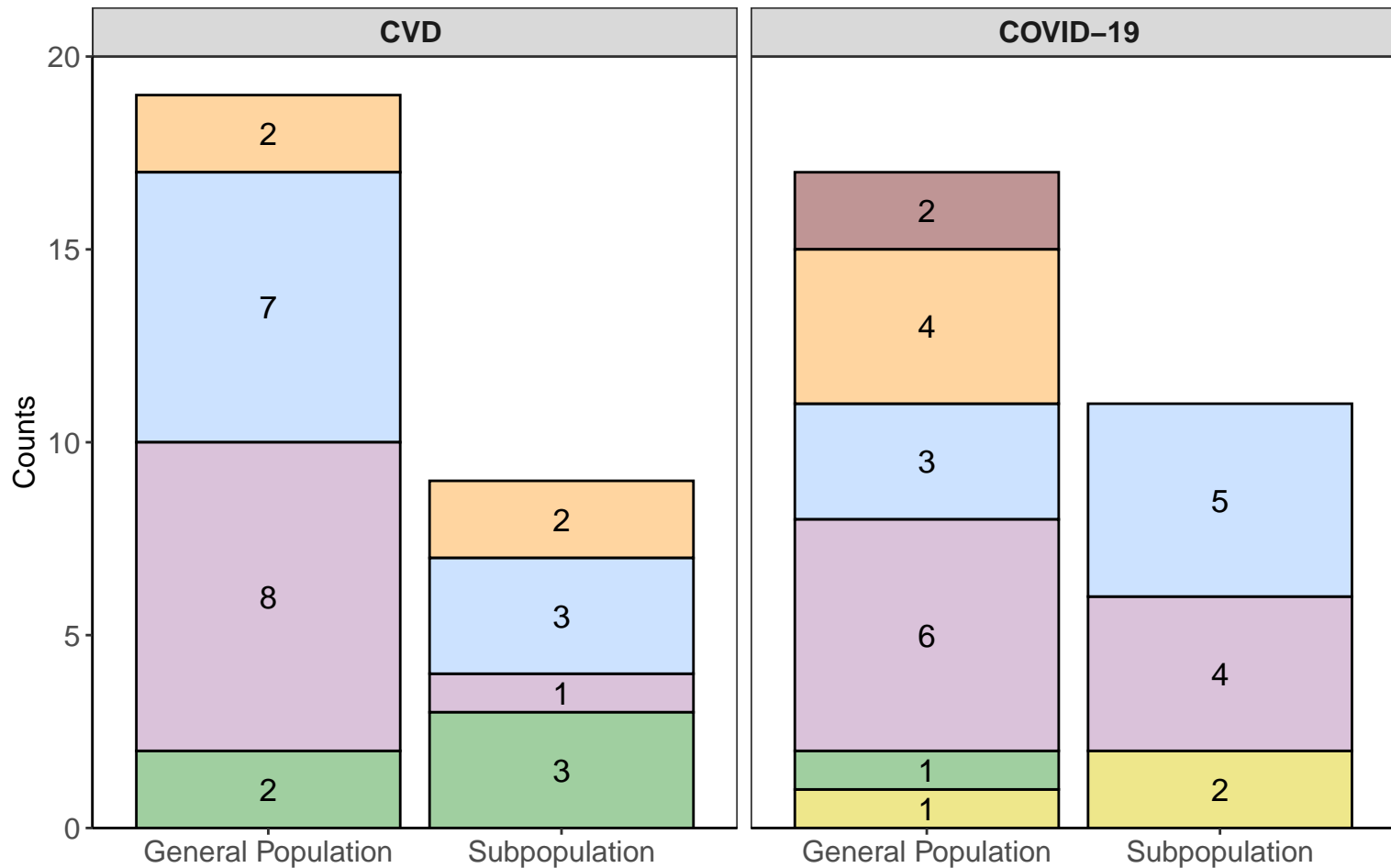

Supplement: Multimedia Appendix 3 [file ojphi_v17i1e66598_app3.pdf]
