# Supplementary material for: Reporting of Fairness Metrics in Clinical Risk Prediction Models Used for Precision Health: Scoping Review
Source: Online J Public Health Inform. 2025 Mar 19;17:e66598. doi: 10.2196/66598 (PMC11966066; doi:10.2196/66598)
Supplement: Multimedia Appendix 4 [file ojphi_v17i1e66598_app4.docx]

| **A 10-question survey for understanding investigators’ barriers for using fairness metrics in clinical risk prediction models** | |
| --- | --- |
| Question 1 | What is the overall goal of the clinical risk prediction model that you are constructing? |
| Question 2 | What were the sensitive features included in your prediction model? |
| Question 3 | What is the intended use of the sensitive features in your prediction models? |
| Question 4 | Are the measures (and how they were collected) consistent with their intended use? |
| Question 5 | How were the sensitive features you selected in Q2 included in your model? |
| Question 6 | What were the criteria used for model evaluation and performance? |
| Question 7 | Were the model evaluation criteria focused on overall performance or the performance within specific subgroups of the data defined by the sensitive variables you chose in Q2? |
| Question 8 | Was model fairness considered and/or assessed? |
| Question 9 | If you answered yes to Q7, please explain how you considered model fairness. If you answered no, please explain what prevented such consideration. |
| Question 10 | If there were any other challenges or insights concerning the use (or non-use) of model fairness in your developed prediction model that you would like to share, please do so below. |

**Supplemental Table 1.** The ten questions included in the questionnaire. All but questions 9 and 10 are multiple choice, with the option to elaborate in a free response.
